# Genome-Wide Identification and Transcriptional Expression Analysis of Annexin Genes in *Capsicum annuum* and Characterization of *CaAnn9* in Salt Tolerance

**DOI:** 10.3390/ijms22168667

**Published:** 2021-08-12

**Authors:** Xiaoxia Wu, Yan Ren, Hailong Jiang, Yan Wang, Jiaxing Yan, Xiaoying Xu, Fucai Zhou, Haidong Ding

**Affiliations:** 1 Joint International Research Laboratory of Agriculture and Agri-Product Safety of Ministry of Education of China, College of Bioscience and Biotechnology, Yangzhou University, Yangzhou 225009, China; xxwu@yzu.edu.cn (X.W.); MZ120181162@yzu.edu.cn (Y.R.); 192101205@yzu.edu.cn (H.J.); mx120200993@yzu.edu.cn (Y.W.); jxyan2018@163.com (J.Y.); xuxiaoying@yzu.edu.cn (X.X.); 2 College of Horticulture and Plant Protection, Yangzhou University, Yangzhou 225009, China

**Keywords:** annexin, bioinformatic analysis, *CaAnn9*, oxidative damage, pepper, salt stress

## Abstract

Annexin (Ann) is a polygenic, evolutionarily conserved, calcium-dependent and phospholipid-binding protein family, which plays key roles in plant growth, development, and stress response. However, a comprehensive understanding of *CaAnn* genes of pepper (*Capsicum annuum*) at the genome-wide level is limited. Based on the available pepper genomic information, we identified 15 members of the *CaAnn* gene family. Phylogenetic analysis showed that CaAnn proteins could be categorized into four different orthologous groups. Real time quantitative RT-PCR analysis showed that the *CaAnn* genes were tissue-specific and were widely expressed in pepper leaves after treatments with cold, salt, and drought, as well as exogenously applied MeJA and ABA. In addition, the function of *CaAnn9* was further explored using the virus-induced gene silencing (VIGS) technique. *CaAnn9*-silenced pepper seedlings were more sensitive to salt stress, reflected by the degradation of chlorophyll, the accumulation of reactive oxygen species (ROS), and the decrease of antioxidant defense capacity. This study provides important information for further study of the role of pepper *CaAnn* genes and their coding proteins in growth, development, and environmental responses.

## 1. Introduction

Annexin (Ann), a subfamily of calcium (Ca^2+^)-dependent and phospholipid-binding protein, is evolutionarily conserved across plants, animals, and microorganisms [1]. Since the first plant Ann was identified from tomato, numerous plant Anns have been discovered [2,3,4,5,6,7]. Plant Ann has the motif or residue of peroxidase, ATPase/GTPase activity, and Ca^2+^ channel regulatory activity, which has many regulatory effects on plant growth and development and stress response [4,7]. Cotton *AnnGh3* overexpression increases the trichome density and length of Arabidopsis leaves [8]. Silencing *AtAnn5*, specifically expressed in mature pollen, results in Arabidopsis abnormal pollen grains and severe male sterility [9]. *GhAnn2* expression is induced by plant hormones IAA and GA3 and downregulated *GhAnn2* expression in cotton inhibits fiber elongation, possibly by regulating Ca^2+^ implantation at the cell apex [10]. The fiber elongation and secondary cell wall biosynthesis are regulated by GhAnnxA [11]. The role of annexins in abiotic and biotic stresses tolerance has been widely studied. In Arabidopsis, AtAnn1 and AtAnn4 interact in a Ca^2+^-dependent manner to regulate tolerance to salt and drought stresses [12]. AtAnn1 level is upregulated by heat treatment and positively regulates the heat-induced increase in [Ca^2+^]cyt and heat tolerance [13]. AtAnn1 also mediates the generation of cold-induced Ca^2+^ signals and mutation of AtAnn1 reduces the magnitude of the cold-induced increase in [Ca^2+^]cyt and consequently decreases freezing tolerance [14]. High temperature induces upregulation of *OsAnn1* in rice, and overexpression of *OsAnn1* in rice regulated ROS production at seedling stage, thereby enhancing heat tolerance [15]. Overexpression of *OsAnn3*, upregulated by ABA and drought stress, confers tolerance to drought by promoting stomatal closure and/or ABA accumulation in rice [16]. Constitutive expression of annexin *BjAnn2* in *Brassica juncea* enhances proline accumulation and maintains ion homeostasis, thereby improving salt tolerance, glucose, and ABA insensitivity of transgenic plants [17]. Arabidopsis with high expression of *AtAnn1* and *AtAnn4* is more resistant to *Meloidogyne incognita*, while *AtAnn1* and *AtAnn4* knockout plants are more sensitive [18]. Annexin8 negatively regulates RPW8.1-mediated cell death and disease resistance in Arabidopsis [19].

In recent years, some progress has been made in the research on the regulation mechanism of plant Anns. AnxGb6 interacts with actin 1, regulating the elongation of cotton fibers [20]. The MYB30 transcription factor responds to oxidative and heat stress through an Ann-mediated [Ca^2+^]_cyt_ signal in Arabidopsis cells [21]. AtAnn4 is involved in the regulation of specific Ca^2+^ signal and the activation of SOS pathway under salt stress. SCaBP8 helps to promote the interaction between AtAnn4 and SOS2, and SOS2 phosphorylation of AtAnn4 protein further enhanced the stability of the AtAnn4–SCaBP8 complex in plants under salt stress [22]. The phosphatase GhDsPTP3a interacts with GhAnn8b and reversely regulates the tolerance of cotton to salt stress [23]. Liu et al. [14] showed that cold activated OST1 which phosphorylates AtAnn1, thereby enhancing its Ca^2+^ transport activity, and further potentiating Ca^2+^ signaling which unraveled a cascade linking OST1-AtAnn1 to cold-induced Ca^2+^ signal generation, which activates the cold response and consequently enhances freezing tolerance in Arabidopsis.

Pepper (*C. annuum* L.) is a globally important horticultural crop and the second largest Solanaceous vegetable worldwide after tomato. Ann genes, however, are yet to be characterized in pepper. In this study, fifteen Ann proteins were identified based on three pepper genome databases. Through genome-wide analysis, the gene structure, gene phylogeny, conserved protein motif, phylogenetic relationship, and the expression pattern under different abiotic stresses and plant hormonal treatments were studied. In addition, the function of *CaAnn9* was further explored. Using the virus-induced gene silencing (VIGS) technique, *CaAnn9*-silenced peppers were more sensitive to salt stress, reflected by aggravated chlorophyll degradation and reactive oxygen species (ROS) accumulation and decrease of antioxidant defense. These results provide critical information about *CaAnn* genes, as well as further identification of the biological functions of *CaAnn* genes in pepper.

## 2. Results

### 2.1. Genome-Wide Identification of Ann in Pepper

To identify the pepper Ann protein family, we performed a BLASTP search of three genome protein sequence databases (zunla v2.0, Chiltepin v2.0, CM334 v.1.55) of *C. annuum* reference genome data using eight Arabidopsis Ann (AtAnn) proteins (Figure S2). The first blast search identified 15, 15, and 12 Ann candidates in the Zunla-1, chiltepin, and CM334 genomes, respectively. In order to further determine the number of Ann candidates in the CM334 database, we searched *C. annuum* cv. CM334 reference genome v1.6 and 17 Ann candidates were identified. To determine the actual number of Ann genes, we designed full-length primers to clone the genes (CAN.G134.113, CAN.G134.117, CAN.G460.1, CAN.G571.6, CAN.G966.1) from CM334 that are not common in the four databases. CAN.G460.1 and CAN.G571.6 could not be cloned, but CAN.G134.113, CAN.G134.117, and CAN.G966.1 could be cloned (data not shown). Therefore, there are also 15 Ann candidates in the CM334 genomes. A total of 15 full-length pepper *CaAnn* genes were ultimately confirmed. These genes were named *CaAnn1*–*CaAnn15* according to their genomic positions in Zunla-1 (Table 1).

As estimated from ExPASy server (http://www.expasy.org/), the CaAnn protein length ranged from 129 AA (CaAnn13) to 341 AA (CaAnn1), and the corresponding open reading frames (ORFs) ranged from 390 to 1026 bp. The MWs ranged from 17.39 to 40.93 kDa, and the theoretical isoelectric point (pI) ranged from 4.99 (CaAnn2) to 9.56 (CaAnn5) (Table 1).

### 2.2. Conserved Regions and Phylogenetic Analysis of CaAnn Proteins

**Figure 1:**
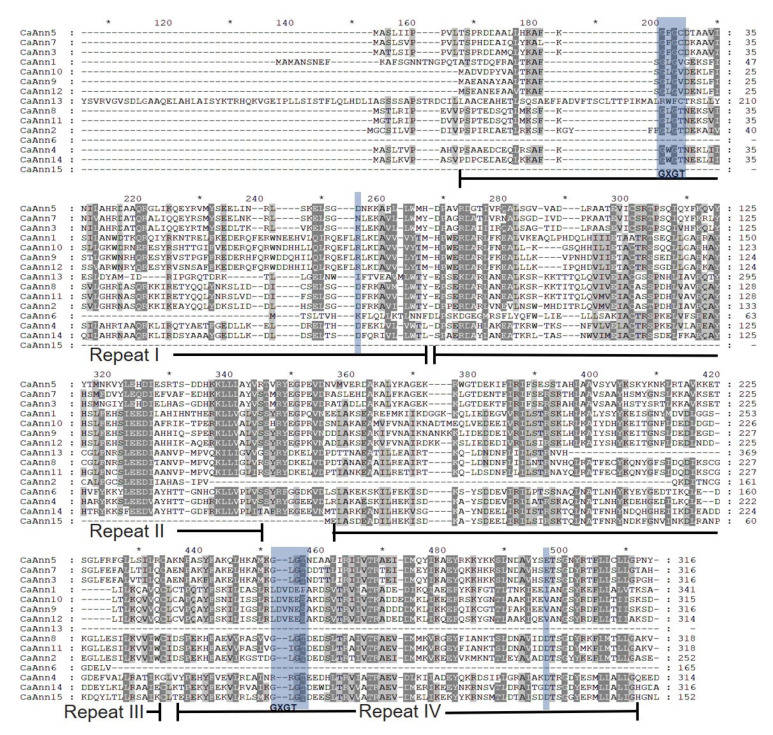
Amino acid sequence alignment of pepper CaAnn proteins. The alignment was performed using Clustal X. The putative annexin repeats (I to IV) are shown below the sequences. The conserved protein sequence (GxGT-[38 residues]-D/E) are shown in blue.

**Figure 2:**
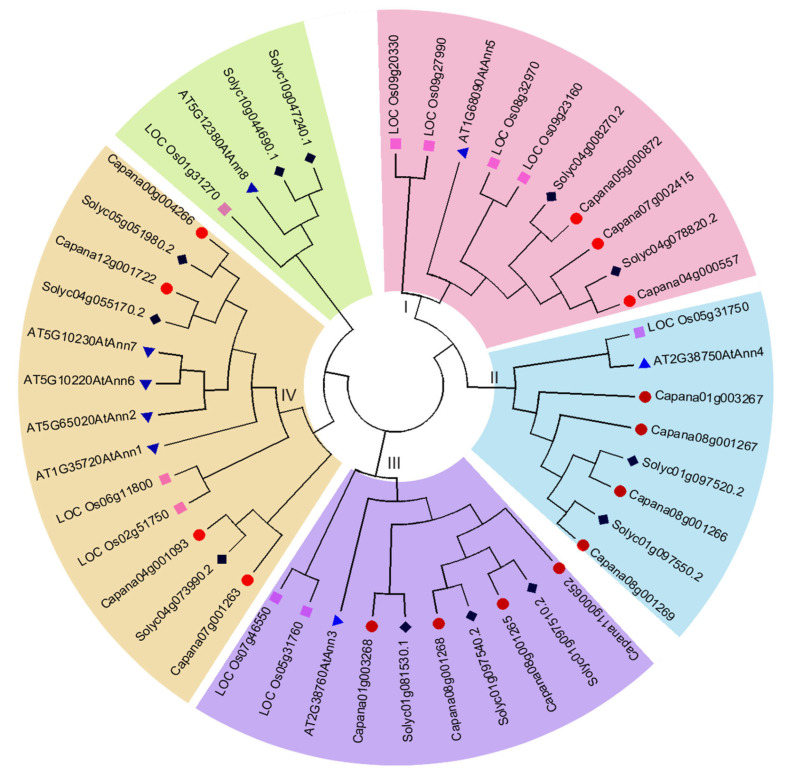
Phylogenetic analysis of pepper CaAnn protein sequences. The 45 amino acid sequences of four plant species were compared with Clustal-W, and the phylogenetic tree was constructed in MEGA-6 using the neighbor-joining method. The roman letters represent the four groups. The different branch colors represent pepper (red), tomato (black), rice (pink), and Arabidopsis (blue).

In order to further explore the conserved regions of CaAnn proteins, MEGA6.0 was used to analyze the protein sequences of 15 members, where highly conserved domains were found (Figure 1). The Ann family should contain Ann repeats. InterPro and Conserved Domain (CD) searches in NCBI were used to verify the existence of annexin repeats in all members. Among the fifteen CaAnn proteins, seven were the typical Ann, which contained four Ann repeats, while only one member contained one Ann repeat and seven members contained two to three Ann repeats (Table 1; Figure 1). In annexins, type II calcium binding sites are determined by the conserved protein sequence (GxGT-[38 residues]-D/E). Here, the conserved sites were found in the first and fourth repeats of pepper, but not in the second and third repeats (Figure 1). At present, the function of some Ann family members in Arabidopsis and rice has been studied [14,16]. To predict the function of CaAnn proteins and the evolutionary relationship between CaAnn proteins and other annexins, a phylogenetic tree using the CaAnn protein sequences of Arabidopsis (8 genes), rice (10 genes), tomato (12 genes), and pepper (15 genes) was constructed based on the neighbor-joining (NJ) criteria of MEGA6.0. All the CaAnn proteins were divided into four clusters (I, II, III, and IV) (Figure 2), which showed that there was a close relationship between the candidate CaAnn proteins in each of the four clusters. Cluster I contained ten members (with one, four, two, and one member of Arabidopsis, rice, tomato, and pepper, respectively). Cluster II included seven members (with one, one, two, and three members of Arabidopsis, rice, tomato, and pepper, respectively). Cluster III contained ten members (with one, two, three, and four members of Arabidopsis, rice, tomato, and pepper, respectively). Cluster IV had thirteen members (with four, two, three, and four members of Arabidopsis, rice, tomato, and pepper, respectively). No CaAnn protein was clustered into the other group (with one, one, and two members of Arabidopsis, rice, and tomato, respectively).

### 2.3. CaAnn Locations and Gene and Protein Structures

According to the pepper genome sequence, 14 *CaAnn* genes were mapped to different chromosomes (Table 1). Five *CaAnn* genes (from *CaAnn8* to *CaAnn12*) are located on chromosome 8. *CaAnn15* could not be located on any chromosome, but on a pseudochromosome named Chr00.

In order to understand the *CaAnn* gene structures, we used the online tool Gene Structure Display Server (GSDS) 2.0 to compare genomic DNA sequences to analyze the structure of exons/introns. The results showed that the *CaAnn* genes of pepper contain 3 to 6 introns, and 7 of the 15 *CaAnn* genes contain 5 introns (Figure S3).

**Figure 3:**
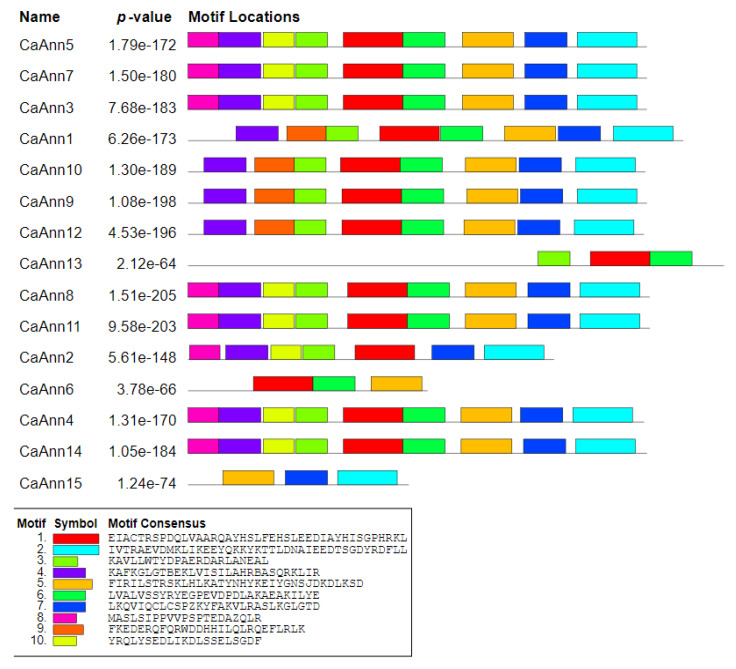
Motif distributions of pepper CaAnn proteins. Ten themes were identified by MEME tool search and are represented by different colors. As described in the methods, Meme online software (Version 5.3.3 http://memesuite.org/tools/meme) was used. The annexin proteins are listed on the left. The different colored boxes represent different motifs and their position in each annexin sequence. The sequences of key motifs are shown on the bottom of the figure.

To further explain the structural diversity of the CaAnn proteins, 10 conserved motifs were predicted using MEME tool (Figure 3). The motifs varied in length from 21 to 41 AA. Most proteins have 8–9 motifs, but three motifs were detected only in three proteins (CaAnn13, CaAnn6, CaAnn15).

### 2.4. Analysis of Cis-Elements in CaAnn Gene Promoter Regions

**Figure 4:**
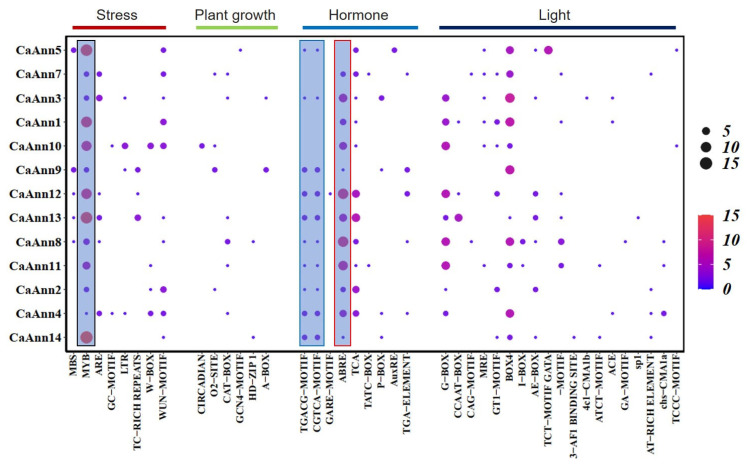
The heat map of cis-element in *CaAnn* promoter regions. The color bar and circle size indicate the number of cis-elements. The black box represents MYB element, the blue box represents MeJA-responsive, and the red box represents ABA responsive module.

To explore the potential function and regulation of *CaAnn* genes identified in the pepper genome, corresponding promoter regions (approximately 2 kb upstream of translation start site) of the 13 CaAnn genes were subjected to PlantCARE online for cis-element analysis. The results showed that 42 types of cis elements were found in addition to the core cis elements, such as TATA box and CAAT box, including 8 types of stress-responsive, 6 types of plant growth, 9 types of phytohormones-responsive and 19 types of light-responsive elements (Figure 4). The light-responsive elements accounted for the largest proportion among the cis-elements. The most common light-responsive motifs were the G-BOX and BOX4 cis-elements. Among the hormone-responsive cis-elements, abscisic acid-responsive elements (ABREs) were the most abundant elements, while CaAnn5 had none of this element. Other cis-elements related to hormones are also found in some *CaAnn* genes, such as methyl jasmonate (MeJA) response elements (TGACG and CGTCA), salicylic acid response elements (TCA), and auxin response elements (TGA elements). However, there are few gibberellin response elements (GARE motif, P-box and TATC-box).

Regarding the cis-element of stress response, MYB was the most abundant element, which was detected in all *CaAnn* genes. In addition, other stress response elements, such as MBS (MYB binding site involved in drought induction), ARE (cis-acting regulatory element essential for anaerobic induction), TC-rich repeating regions (related to defense and related to stress-response element), LTR (low temperature response element), W-box (fungal elicitor response element), and WUN motif (wound stress-response element) are also present in some *CaAnn* genes. The results here suggested that *CaAnn* genes might play a role in transcriptional control of plant growth, hormones, and stress responses.

### 2.5. Expression Profile of CaAnn Genes in Different Tissues

**Figure 5:**
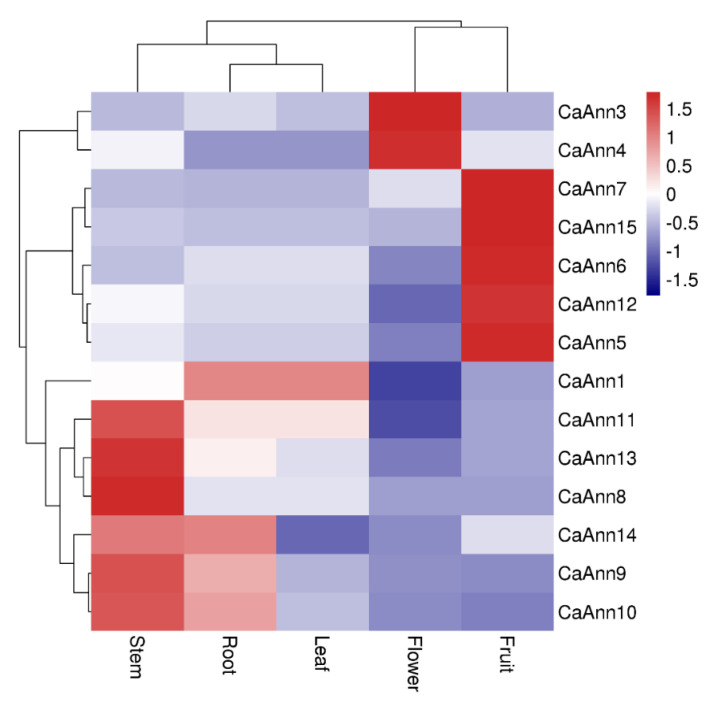
Tissue-specific expression analysis of pepper *CaAnn* genes. Total RNA was extracted from different tissues (root, stem, leaf, flower, and green fruit) and used for real-time quantitative PCR. The expression level was normalized against pepper *actin 97* (NCBI LOC107870208) and Phosphoenolpyruvate carboxylase genes (*PC2*, NCBI LOC107854728). Each value represents the average relative expression level of the three replicates. The bar in the upper right corner represents the expression value of log2, and different colors represent different expression levels. Red indicates relatively high expression and blue indicates relatively low expression.

*CaAnn* may be involved in plant growth and development, and its expression pattern in different organs of pepper may reflect this process. To address this, their expression profiles in five different tissues of pepper cultivar xinsujiao15 (root, stem, leaf, flower, and green fruits) were analyzed through qRT-PCR. The expression pattern of each *CaAnn* gene was different in various tissues (Figure 5). For vegetative organs, the *CaAnn1* transcript level was the highest in leaves, followed by *CaAnn11*. In stems, *CaAnn8* showed the highest transcript level. In contrast, the transcription level of *CaAnn*14 was the highest in roots. For flower and fruit, *CaAnn3* and *CaAnn4* exhibited the highest transcript level in the flowers, whereas *CaAnn5*, *CaAnn6*, *CaAnn5*, *CaAnn12*, and *CaAnn15* exhibited the highest transcript levels in the fruits. These results indicated that *CaAnn* genes might be involved in pepper growth and/or development.

### 2.6. Expression Profile of CaAnn Genes in Response to Abiotic Stress and Hormonal Treatment

**Figure 6:**
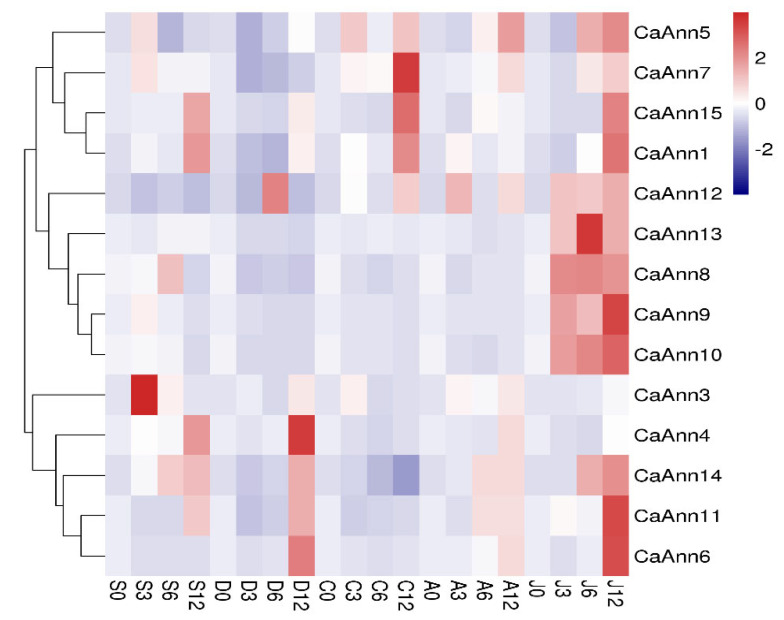
Expression patterns of *CaAnn* genes under abiotic stresses and plant hormone treatments. One-month-old pepper seedlings were treated with salt (200 mM NaCl), drought (15% *w*/*v* PEG6000), cold (4 °C), ABA (100 μM), and MeJA (100 μM) for 0, 3, 6, and 12 h. Total RNA was extracted from leaves and used for real-time quantitative PCR. The expression level was normalized against pepper *actin 97* and *PC2* genes. Each value represents the average relative expression level of the three replicates. The bar in the upper right corner represents the expression value of log2, and different colors represent different expression levels. Red indicates relatively high expression and blue indicates relatively low expression.

The promoter analysis of *CaAnn* genes suggested that these genes are involved in the response of pepper to different stresses. In this study, the *CaAnn* transcriptional expressions were analyzed by qRT-PCR after salt, drought, cold, and exogenous ABA and MeJA (Figure 6). In all treatments, almost all *CaAnn* genes showed expression patterns under these challenges, and only one gene, *CaAnn2*, could not be detected in leaves. Under salt treatment, the transcript levels of ten *CaAnn* genes were upregulated and two *CaAnn* genes (*CaAnn6*, *CaAnn12*) were downregulated. The *CaAnn3* gene expression was highly induced at 3 h after salt stress. Seven *CaAnn* genes showed upregulation at 12 h post drought stress, and the expression level of *CaAnn4* was the highest. For the cold inoculation, five gene expressions (*CaAnn1*, *CaAnn5*, *CaAnn7, CaAnn12*, *CaAnn15*) were upregulated, however, others showed a downward trend. The *CaAnn7* expression was the highest at 12 h after cold stress. The selected *CaAnn* genes above were also exposed to hormonal stress (ABA and MeJA). The results showed that eight CaAnn genes were upregulated at different time points after ABA treatment, but twelve CaAnn genes were significantly responsive to MeJA and five (*CaAnn8*, *CaAnn9*, *CaAnn10*, *CaAnn12*, *CaAnn13*) were continuously activated by MeJA.

### 2.7. CaAnn9 Is Involved in Salt Tolerance

Previously, we screened this gene family through proteomics technology, and only the protein expressed by this gene could significantly respond to *Bemisiatabaci* stress and JA, and had the highest homology with Arabidopsis gene AtAnn4 (Figure S4). Recently, AtAnn4 has been proved to be involved in salt stress-induced Ca^2+^ increase [22]. Here, the function of *CaAnn9* under salt stress was determined.

**Figure 7:**
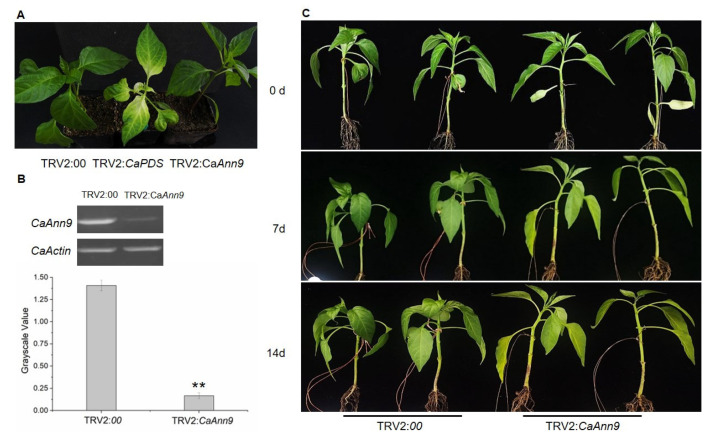
*CaAnn9* silenced in pepper decreased tolerance to salt stress. (**A**) The phenotype of *CaAnn9*-silenced seedlings. Photographs were taken 40 days post-infiltration. TRV2:*CaPDS* was used as a positive control vector. TRV2:*00* was used as a control vector. (**B**) Silencing efficiency of *CaAnn9* in *CaAnn9*-silenced plants. Expression of *CaAnn9* in *CaAnn9*-silenced plants. RT-PCR analyses of the expression of *CaAnn9* are shown in the three independent plants. *CaActin* was used as an internal control. Grayscale value was measured by ImageJ1.51 j8 [24]. ** Represents significant differences at *p* ≤ 0.01. Error bars represent SD values of five bands. (**C**) Phenotype of *CaAnn9*-silenced and control plants under 200 mM NaCl. The *CaAnn9*-silenced and control seedlings were treated with 200 mM NaCl for 7 d and 14 d.

First, we found that the expression level of *CaAnn9* was induced at 3 h after salt stress and decreased significantly at 6 and 12 h (Figure 6), indicating that *CaAnn9* was involved in salt stress. Secondly, to verify this assumption, we silenced *CaAnn9* using a virus-induced gene silencing (VIGS) method in pepper. The positive control vector (TRV2: CaPDS) was used to silence the pepper *CaPDS* gene. After silencing, the leaves showed a photobleaching phenotype. The negative control group was TRV2:00, showing no difference in visual phenotype (Figure 7A). When TRV2:*CaPDS* plants showed a photobleaching phenotype, the silencing efficiency of TRV2:*CaAnn9* and TRV 2:00 was detected, which was over 85% (Figure 7B). Subsequently, *CaAnn9*-silenced and control plants were used for further salt treatment.

**Figure 8:**
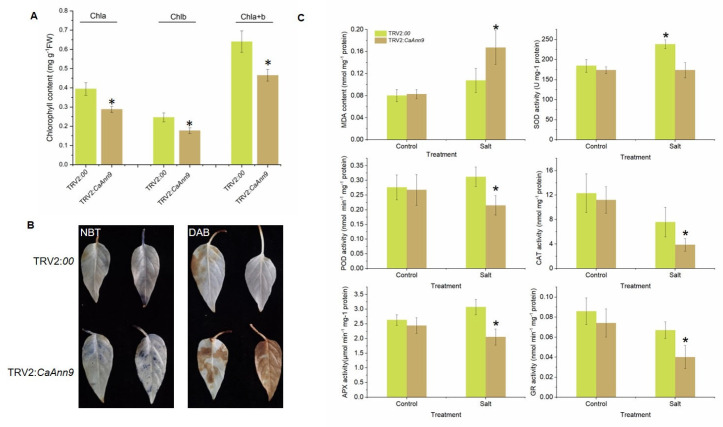
The oxidative damage in *CaAnn9-*silenced plants under salt stress. (**A**) The content of chlorophyll a (Chla), chlorophyll b (Chlb), and total chlorophyll (Chla+b) in the leaves of *CaAnn9-*silenced seedlings after 7 days 200 mM NaCl stress. (**B**) NBT and DAB staining in leaves of *CaAnn9*-silenced plants after 7 days of 200 mM NaCl stress. (**C**) The activities of ROS-scavenging enzymes in the leaves of *CaAnn9*-silenced plants after 7 days of 200 mM NaCl stress. Membrane lipid peroxidation products MDA, SOD, POD, CAT, APX, and GR activities were determined. * Represents statistically significant values at *p* ≤ 0.05. Error bars represent SD values of three replicates.

The *CaAnn9*-silenced and control pepper seedlings were treated with 150 mM NaCl for 7 and 14 days. Before salt stress, there was no significant difference in visual phenotype. After 7 days of treatment, however, the *CaAnn9*-silenced plants showed symptoms and the leaves turned yellow, while the control plants only turned a little yellow. After 14 days of treatment, the *CaAnn9*-silenced plants turned more yellow (Figure 7C). Therefore, the physiological parameters were measured after 7 days. *CaAnn9*-silenced plants had lower chlorophyll a, chlorophyll b, and total chlorophyll than the control (Figure 8A). In order to evaluate the effect of *CaAnn9* on ROS accumulation, the H_2_O_2_ and O_2_^.-^ of pepper plants under salt stress were detected by NBT and DAB staining. After 7 days of salt treatment, the NBT and DAB staining areas in the *CaAnn9*-silenced leaves were significantly higher than those in the control (Figure 8B). The change of ROS accumulation was reflected in the accumulation of MDA. The membrane lipid peroxidation product (MDA) content was significantly higher in the *CaAnn9*-silenced leaves than in the control (Figure 8C). In addition, the antioxidant defense enzyme system was checked. As shown in Figure 8C, the *CaAnn9*-silenced plants had lower activities of SOD, POD, CAT, APX, and GR than the control.

## 3. Discussion

By now, a large number of plant gene families have been found, such as Arabidopsis, tomato, rice, and maize [5], and most of the functional studies of Anns are mainly focused on Arabidopsis, followed by rice. However, little is known about the identification and characterization of the Ann gene family in pepper. Here, a genome-wide identification showed 15 *CaAnn* genes from ‘Zunla-1′, ‘CM334′, and ‘Chiltepin’ databases of the pepper genome named *CaAnn1*-*CaAnn15* (Table 1). In the past, it has been proposed that the plant *Ann* gene family was small and the diversity was low. Among the better-analyzed plant annexin families, Arabidopsis has 8 *Anns*, and rice has 10 expressed *Anns* [25,26]. Recently, however, 23, 25, and 26 annexin genes were identified from soybean, wheat, and B. napus genome databases, respectively [5,27,28]. In the present study, the 15 *CaAnn* gene number was very similar to that in *Gossypium raimondii* and *Vitis vinifera* [29]. It was speculated that all *Anns* have evolved from a common ancestor, and gene duplication events might lead to an increase in the number of genes, which could be seen from the similarity of amino acid sequences and their genomic location [7]. Based on phylogenetic analysis of 45 CaAnn proteins from pepper, Arabidopsis, tomato, and rice, the 15 CaAnn proteins were divided into five groups, which was consistent with a previous report on maize [30]. However, Ann proteins are divided into six groups in wheat [27] and Brassicaceae species [5]. In fact, according to the existing phylogenetic analysis of plant Ann gene grouping, Ann proteins can be divided into four groups according to Arabidopsis genome: AtAnn3, AtAnn4, AtAnn5, and others (AtAnn1, 2, 6, 7, 8). Sometimes, AtAnn8 can be a separate group. Here, for example, the group with AtAnn4 is CaAnn1, 9, 10, 12 (Figure 2). This suggested that the CaAnn proteins clustered in the same group might have similar biological functions.

Plant Anns have been detected in different organs and the expression appears to be linked to growth and development [27,29]. Here, several *CaAnn* genes in pepper showed preferential expression patterns in organs, indicating their specific or important roles. The expression levels of five *CaAnn* genes were expressed in roots (Figure 5). For example, *CaAnn1* and *CaAnn14*, orthologous to *AtAnn4* and *AtAnn1*, respectively, were highest expressed in roots. The expression levels of *AtAnn1* and *AtAnn2* are consistent with their roles in root growth and development [31,32]. *TaAnn1* and *TaAnn2* were expressed preferentially in the root [27]. *AtAnn5* was specifically expressed in mature pollen, promoted the reproductive development of *A. thaliana*, and was necessary for pollen and embryo formation [33]. The genes of *B. napus* homologous to *AtAnn5* were mainly expressed in buds and new pistils [5]. In this study, only two genes, *CaAnn3* and *CaAnn4*, were preferentially expressed in flowers (Figure 7c). *CaAnn3* shared high identity with subgroups *AtAnn5*, suggesting it might be involved in the development or maturation of floral organs. Furthermore, five *CaAnn* genes (*CaAnn*5, 6, 7, 12, 15) were observed mainly in fruits (Figure 5). Proust et al., found an annexin special expression during the early and ripening stages of pepper fruit development by using Northern blot analyses [34]. The expression of strawberry annexins *FaAnn5a* and *FaAnn8* increased during the whole progression of development of strawberry fruit [35]. In pepper, we can focus on the function of CaAnn3 and CaAnn4 in pepper flower development.

Previous studies have shown that there are cis elements in the annexin gene that can respond to various stresses [25,27,36]. All pepper *CaAnn* genes contain MYB-motif elements, which indicated that *CaAnn* genes could response to different abiotic stresses, reflected by changes under drought, salt, and cold (Figure 6). ABA is a plant hormone that responds to abiotic stress, so exogenous application of ABA can simulate the effects of stress conditions [37]. Here, all except one gene, *CaAnn5*, had ABRE, and eight genes were induced by exogenous ABA (Figure 6), which was similar to other plant *Ann* genes reported [27]. Li et al. [16] confirmed that the expression of *OsAnn3* was induced by ABA and drought stress. Overexpression of rice *OsAnn3* conferred drought tolerance by promoting stomata closure and/or ABA accumulation. In this study, most *CaAnn* members contain MeJA-responsive cis-elements (Figure 4) and all *CaAnn* genes except *CaAnn3* and *CaAnn4* could be induced by exogenous MeJA. Up to now, there are few studies on plant Anns involved in JA-mediated signaling. Our previous studies [38] showed that the CaAnn9 protein level was induced by *Bemisia tabaci* and MeJA, where there were significant differences in CaAnn9 expression between resistant and sensitive peppers, indicating that CaAnn9 played an important role in plant tolerance to *B. tabaci*. He et al. [5] showed that *BnAnn* genes in a light green module were involved in JA signaling response in *B. napus*. Zhao et al. [18] reported the involvement of AnnAt1 and AnnAt4 in *A. thaliana* responding to *Meloidognye incognita*. As a novel effector, MiMIF-2 could interact with annexin to manipulate host immune response.

CaAnn9 was of concern because it expressed the highest protein abundance in response to the *Bemisiatabaci* stress shown in our previous study [38]. CaAnn9 was homologous to AtAnn4, which is one of the most homologous proteins in pepper. AtAnn4 was a predicted calcium permeability transporter. The processes of AtAnn4 and AtAnn1 are regulated by their binding properties [12]. Plants lacking *atann4* showed a variety of defects, including less increase of [Ca^2+^]_cyt_ than wild-type plants under various stress conditions [21]. In pepper, the expression level of *CaAnn9* was the highest in stem and the lowest in fruit (Figure 5). *CaAnn9* could response to salt and JA (Figure 6). However, the specific function of the CaAnn9 is still unclear. Recently, AtAnn4 were identified in mediating calcium transients upon salt stress [22]. Therefore, the function of *CaAnn9* in response to salt was examined in the present study. It was observed that *Ann9* expression could be induced in the early stage of salt stress. In order to verify whether the *CaAnn9* gene was related to salt stress, we adopted the VIGS method. Under normal conditions, there was no phenotypic difference between *CaAnn9*-silenced plants and control plants (Figure 7). Under salt stress, *CaAnn9*-silenced plants were more sensitive to salt stress, compared with control plants.

Arabidopsis AtAnn4 and AtAnn1 have been shown to be a positive regulator of salinity tolerance [12,21,22]. The salt tolerance of plants is usually related to the enhancement of the antioxidant defense system. Under different stresses and intensities, there are differences in the antioxidant enzyme activities, which has also been observed in other botanical families, such as Malvaceae [11] and Poaceae [15]. In this study, it was observed that the accumulation of ROS and MDA in *CaAnn9*-silenced plants was significantly higher than that of control plants (Figure 8B,C), while the activities of SOD, POD, CAT, APX, and GR were lower in CaAnn9-silenced plants than control plants (Figure 8C). The results suggested that CaAnn9-mediated oxidative stress tolerance, caused by salt stress, was through the regulation of antioxidant enzymes. This correlation has also been observed with other Ann transgenes [17,39]. For example, under stress conditions, the overexpression of *OsAnn5* enhanced the tolerance to abiotic stress by effectively eliminating ROS and balancing the expression of SOD and CAT antioxidant enzymes. Anns-mediated salt-induced [Ca^2+^]_cyt_ elevation is of great significance. However, the underlying molecular mechanism is still unclear. AtAnn1 might act as a Ca^2+^ permeability transporter, mediating the increase of [Ca^2+^]_cyt_ induced by ROS- and NaCl [1,32]. Recently, Ma et al. [22] found that under salt stress, AtAnn4 played a key role in the generation of calcium signals, activating the SOS pathway in Arabidopsis. SCaBP8 helped to promote the interaction between AtAnn4 and SOS2, and SOS2 phosphorylation of AtAnn4 protein further enhanced the stability of the AtAnn4-SCaBP8 complex in plants under salt stress. In cotton, through disrupting GhANN8b-mediated SOS1 upregulation, GhDsPTP3a reduced Na^+^ efflux. In addition, GhDsPTP3 might also regulate the dephosphorylation of proteins responsible for Na^+^ flux to prevent Na^+^ efflux, such as SOS1 and CIPK24 [23]. The specific mechanism of CaAnn9 under salt stress needs to be further explored

## 4. Materials and Methods

### 4.1. Identification of Pepper Annexins

The eight AtAnn proteins were retrieved from the *A. thaliana* Araport11. These sequences were subjected to BLASTp searches in the three genome protein sequence databases (zunla v2.0, CM334 release1.55, Chiltepin v2.0) of *C. annuum* Genome Data (https://solgenomics.net/organism/Capsicum_annuum/genome accessed on 15 May 2021) using the blastp program in BLAST and the E-value cut-off was set as 1e-10. In these databases, “annexin” was also used as the keyword for homologous searches. The genomic regions, transcripts, and products are from Pepper Zunla 1 Ref_v1.0 Primary Assembly (https://www.ncbi.nlm.nih.gov/genome/?term=txid4072 accessed on 15 May 2021).

The Ann repeats were characterized using InterPro (http://www.ebi.ac.uk/interpro accessed on 10 June 2021). The Compute pI/Mw tool (http://web.expasy.org/compute_pi/ accessed on 10 June 2021) was used to calculate the molecular weight (Mw) and isoelectric point (pI). Gene structure display server (GSDs) was used to analyze the exon and intron structure of the Ann genes (http://gsds.cbi.pku.edu.cn accessed on 10 June 2021). The 2.0-kb sequences upstream of Ann genes were analyzed by PlantCARE (http://bioinformatics.psb.ugent.be/webtools/plantcare/html/ accessed on 15 June 2021).

### 4.2. Phylogenetic Analysis

The Ann sequences of tomato, Arabidopsis, and rice were downloaded from databases *S. lycopersicum* ITAG3.2, *A. thaliana* Araport11, and *O. sativa* v7.0, respectively. Multiple sequence alignments of Anns were performed using ClustalX (http://www.clustal.org/clustal2/ accessed on 20 June 2021) and the tree was constructed by the neighbor-joining (NJ) method with MEGA 6.0 with 1000 bootstrap replications.

### 4.3. Motif Analysis of CaAnn Proteins

The MEME online program (Version 5.3.3 http://meme-suite.org/ accessed on 10 June 2021) identified different motifs in pepper annexin protein sequences. The parameters of MEME were as follows: number of repetitions, any; maximum number of motifs, 10; optimum width of each motif, between 6 and 50 residues.

### 4.4. Plant Materials and Treatments

Pepper cultivar xinsujiao No5 was used in this study. Pepper seedlings were grown in a greenhouse at 28 °C with a 16 h light/8 h dark photoperiod. The relative humidity was controlled at approximately 50%. To investigate the organ-specific expression pattern, different organ samples including roots, stems, and leaves were collected from 6-week-old seedlings. Flowers and fruits were collected from mature plants. Four-week-old seedlings in similar growth conditions were used for abiotic and hormone treatments. To simulate high temperature and cold stresses, pepper seedlings were transferred to incubators at 42 °C and 4 °C (16 h light/8 h dark photoperiod), with the seedlings grown at 28 °C as control. Seedlings were treated with 200 mM NaCl solution as salt stress, and 15% PEG (*w*/*v*) solution was used to simulate drought stress. For hormone treatments, seedlings were incubated in 100 μM abscisic acid (ABA) and 100 μM methyl jasmonate (MeJA), respectively, while the control seedlings were mock-treated with water. The leaves were collected at 0 h, 3 h, 6 h, and 12 h for each treatment. All the samples above were immediately frozen in liquid nitrogen and stored at −80 °C for follow-up.

### 4.5. Expression Analysis of CaAnn Genes

Total RNA was extracted using TriZol reagent (Takara Bio, Shiga, Japan) according to the instructions. Both RNA concentration and quality were assayed by a UV spectrophotometer (Table S1) and gel electrophoresis (Figure S1). About 1.0 μg of total RNA of every sample was used for synthesizing the first strand complementary DNA (cDNA) with SuperScript double stranded-cDNA synthesis kit. qPCR SYBR Premix Ex Taq (Takara) was used to conduct qRT-PCR analysis. The PCR reaction system was 20 μL. Triplicate measurements were performed. The *actin 97* (NCBI LOC107870208) and phosphoenolpyruvate carboxylase 2 (*PC2*, NCBI LOC107854728) were used as internal control. The gene relative expression levels were evaluated by the 2^−ΔΔCt^ method (Table S2) [40]. The primers used are shown in Table S3.

### 4.6. Virus-Induced Gene Silencing (VIGS) of Pepper CaAnn9 Gene

The pTRV1 (tobacco rattle virus 1) and pTRV2 plasmids were kindly provided by Prof. Ouyang (College of Horticulture and Plant Protection, Yangzhou University). The pTRV2-CaPDS/pTRV2-CaAnn9 were constructed by digestion and ligation of the pepper gene *CaPDS* (NM_00324813.1)/*CaAnn9* (Capana08g001266) fragment into the corresponding site in pTRV2 vectors (the primers are shown in Table S3), and placed under the control of the cauliflower mosaic virus CaMV35S promoter. The recombinant plasmids were transformed into *A. tumefaciens* strain GV3101, which were cultured in LB media (containing 50 mg l^−1^ kanamycin, 50 mg L^−1^ rifampicin, 10 mM MES, 20 μM acetosyringone) on shaking incubator (250 rpm) at 28 °C for 14–16 h. When the agrobacterium liquid reached OD_600nm_ = 0.6, the GV3101 cells were harvested by being centrifuged at 3000 rpm for 20 min, then were resuspended with infiltration buffer (10 mM MgCl_2_, 10 mM MES, 200 μM acetosyringone) to a final OD_600nm_ 0.8–1.0. The suspension liquid was kept in the dark and were incubated at room temperature for 3 h before inoculation.

Equal volumes of TRV1 and TRV2, TRV1 and TRV2-*CaPDS*, TRV1 and TRV2-*CaAnn9* Agro cultures were mixed respectively, and introduced into 2-week-old pepper leaves using a 1 mL needless syringe. The incubated seedlings were grown in the dark for 48 h and then transferred to 24 °C growth chamber for 3–4 weeks until the TRV2-CaPDS infiltrate seedlings showed photobleaching symptoms. RT-PCR was performed to detect the transcript level of the *CaAnn9* gene in TRV2-CaAnn9-inoculated seedlings.

The seedlings that showed better gene silence level by RT-PCR were selected and inoculated in 200 mM NaCl solution. The leaves 7 d and 14 d after treatment were harvested for further physiological experiments.

### 4.7. Chlorophyll Content

The chlorophyll (Chl) content in the disc leaves was measured spectrophotometrically, which was carried out after extraction of the leaf discs with 95% ethanol.

### 4.8. Oxidative Damage and Antioxidant Enzyme Activities

According to the method of Zhang et al. [41], the superoxide radicals (O_2_^•−^) and H_2_O_2_ were visually detected in pepper leaves by using nitroblue tetrazolium (NBT) and 3,3-Diaminobenzidine (DAB) as a substrate, respectively. Malondialdehyde (MDA) content was determined to estimate lipid peroxidation, and MDA content was determined by thiobarbituric acid (TBA) reaction according to Jiang and Zhang [42]; MDA content was calculated using the extinction coefficient of 155 mM^−1^ cm^−1^.

The antioxidant enzyme activities were measured according to the study of Ding et al. [43]. Total superoxide dismutase (SOD) activity was assayed through monitoring the inhibition of photochemical reduction of nitroblue tetrazolium. One unit of SOD activity was defined as the amount of enzyme required to cause 50% inhibition of the reduction of NBT monitored at 560 nm. POD activity was measured using guaiacol as a substrate. CAT activity was determined by following the consumption of H_2_O_2_ (extinction coefficient 39.4 mM^−1^cm^−1^) at 240 nm. APX activity was determined by following the decrease of ASC by H_2_O_2_ in A290 (extinction coefficient 2.8 mM^−1^cm^−1^).

### 4.9. Statistical Analysis

All experiments were performed and analyzed with at least three biological replicates. The data were expressed as an average ± standard deviation (SD). A statistical comparison was made using one-way repeated measures analysis of variance (ANOVA), followed by Fisher’s least significant difference (LSD) analysis. *p*-values ≤ 0.05 were considered to be significantly different.

## 5. Conclusions

This study is the first systematic analysis of pepper annexin. Fifteen CaAnn proteins were identified and divided into four subgroups. Genome wide bioinformatics analysis was performed to study phylogeny, gene structure, conserved protein motif, and promoter-cis acting elements. *CaAnn* genes showed different tissue-specific and abiotic stress response expression patterns, which indicated that they might play a specific role in different tissue responses to different stresses. Importantly, *CaAnn9* showed positive regulation of salt tolerance through activation of major antioxidant enzymes controlling ROS metabolism balance. The present study provided the key information about pepper *CaAnn* genes and their encoded proteins, as well as further research on CaAnn proteins, especially on CaAnn9.

## Figures and Tables

**Table 1:** List of *annexin* genes in pepper.

Gene ID Zunla v2.0	Gene Name	Chr	CDS (bp)	AA	pI	Mw (kD)	Annexin Repeats	Annotation ID1 Chiltepin v2.0	Annotation ID2 CM334 v.1.55	Annotation ID3 CM334v.1.6
Capana01g003267	CaAnn1	1	1026	341	6.58	39.18	2	Capang08g000204	CA01g26520	CAN.G1249.15
Capana01g003268	CaAnn2	1	759	252	4.99	28.26	3	Capang08g000203	CA01g26530	CAN.G1249.14
Capana04g000557	CaAnn3	4	951	316	9.16	35.44	4	Capang04g000505	CA04g18590	CAN.G1030.12
Capana04g001093	CaAnn4	4	945	314	5.71	35.85	4	Capang04g001032	CA00g85100	CAN.G1836.3
Capana05g000872	CaAnn5	5	951	316	9.56	35.95	4	Capang00g002303		CAN.G966.1
Capana07g001263	CaAnn6	7	498	165	5.96	19.06	2	Capang07g001288	CA07g10540	CAN.G834.5
Capana07g002415	CaAnn7	7	951	316	7.18	35.66	4	Capang07g002413	CA07g20670	CAN.G623.41
Capana08g001265	CaAnn8	8	957/975	318/324	6.47	35.82	4	Capang01g003847		CAN.G134.113
Capana08g001266	CaAnn9	8	951	316	6.28	36.27	3	Capang01g003848	CA08g10470	CAN.G134.114
Capana08g001267	CaAnn10	8	948	315	5.81	36.29	3	Capang01g003849	CA08g10480	CAN.G134.115
Capana08g001268	CaAnn11	8	957	318	6.91	35.8	4	Capang01g003850	CA08g10490	CAN.G134.116
Capana08g001269	CaAnn12	8	945	314	6.39	36.26	3	Capang01g003851		CAN.G134.117
Capana11g000652	CaAnn13	11	390/459	129/152	7.11	40.93	1	Capang11g000587	CA11g14100	CAN.G84.41
Capana12g001722	CaAnn14	12	951	316	5.36	36.14	4	Capang00g001945	CA00g83440	CAN.G1795.1
Capana00g004266	CaAnn15		459	152	5.51	17.39	2	Capang00g00115	CA05g13700	CAN.G151.32

## Data Availability

The data supporting the results of this study can be obtained in the Supplementary Materials of this article and can be obtained from the corresponding author upon reasonable request.

## Supplementary Materials

The following are available online at https://www.mdpi.com/article/10.3390/ijms22168667/s1.
