# Supplementary material for: Genome-Wide Identification and Transcriptional Expression Analysis of Annexin Genes in Capsicum annuum and Characterization of CaAnn9 in Salt Tolerance
Source: Int J Mol Sci. 2021 Aug 12;22(16):8667. doi: 10.3390/ijms22168667 (PMC8395446; doi:10.3390/ijms22168667)
Supplement: Supplementary file 1 [file ijms-22-08667-s001.zip › Supplementary fIgures .pdf]

Supplementary figures

Sample: 1-17

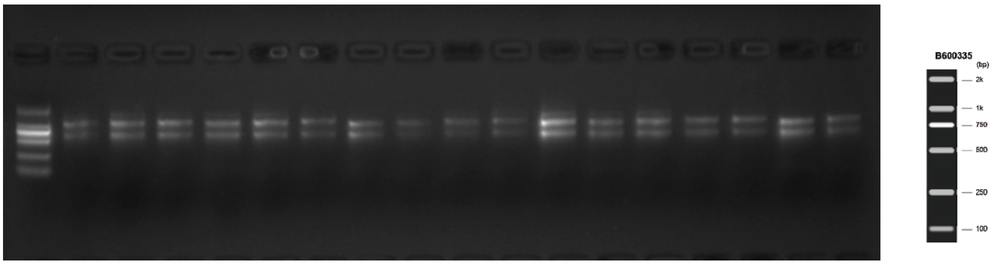

Sample: 18-24

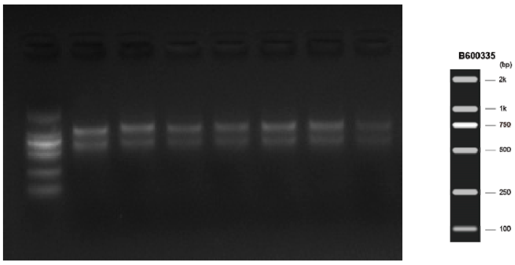

Figure S1 Electropherograms of RNA extract from 24 samples. Samples 1-5 were the total RNA extracted from different tissues (leaves, green fruit, flower, root and stem). Sample 6-9 were the total RNA extracted from pepper leaves in different times (0, 3, 6 and 12 h) without treatment. Samples 10-12, 13-15, 16-18, 19-21, 22-24 were extracted from pepper leaves treated with salt (200 mM NaCl), drought (15% w/v PEG6000), cold (4°C), ABA (100 μM), and MeJA (100 μM) for 3, 6 and 12 h respectively.

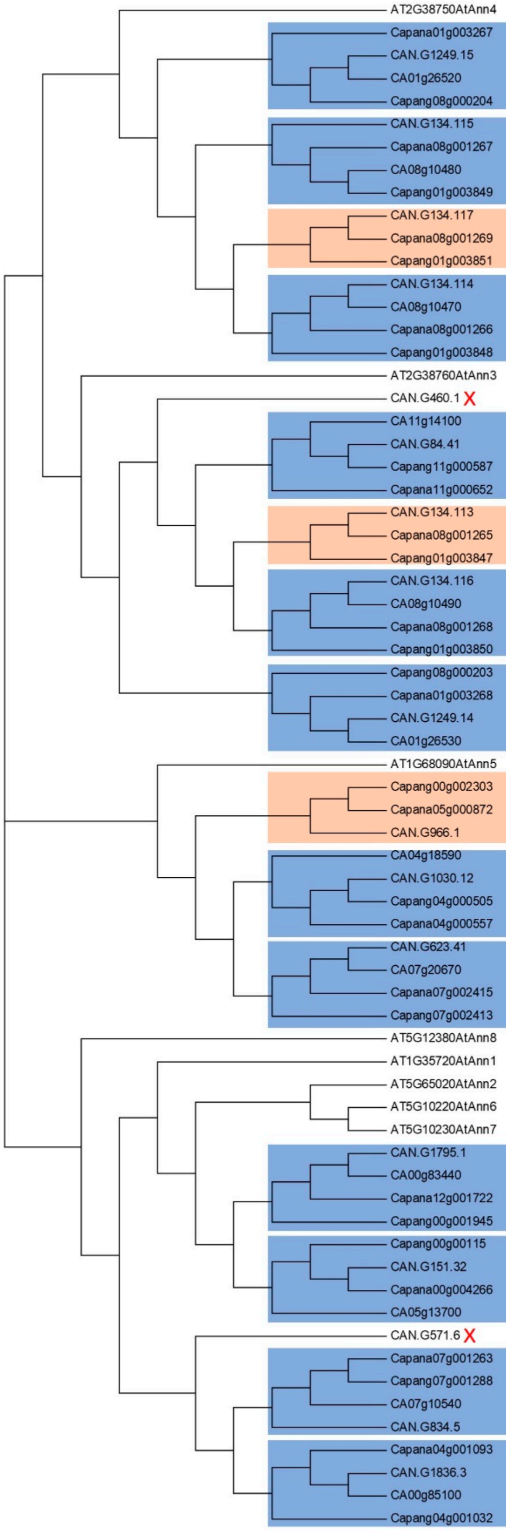

Figure S2 Phylogenetic analysis of pepper CaAnn protein sequences from four genome databases The amino acid sequences of 45 full-length protein sequences from 4 plant species were aligned with Clustal W, and the phylogenetic tree was constructed in MEGA-6 using the neighbor-joining method.

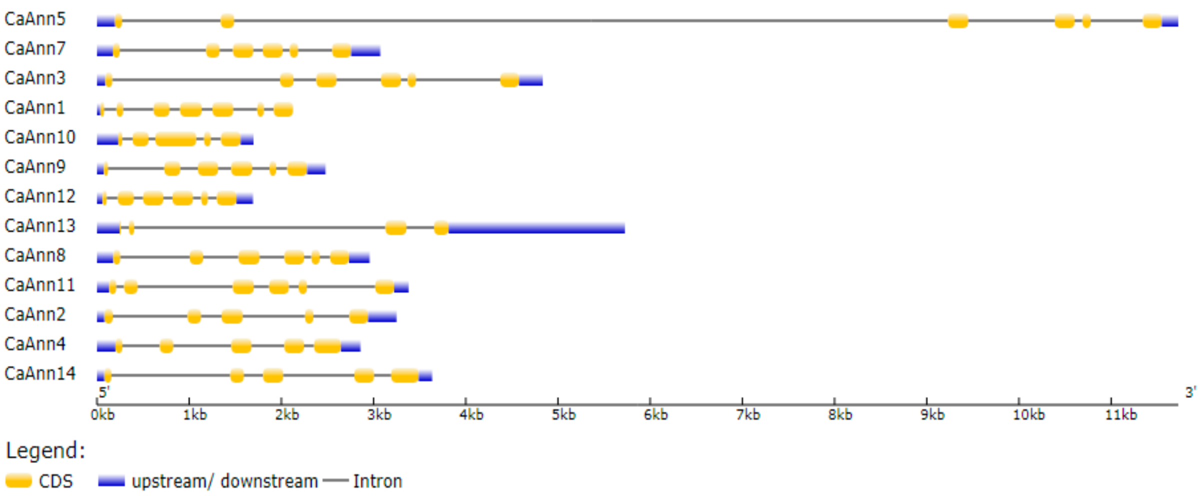

Figure S3. Gene structure of pepper *CaAnn* genes. Gene structures were generated from GSDS (http://gsds.cbi.pku.edu.cn/). Introns and exons are represented by black lines and orange boxes, respectively.

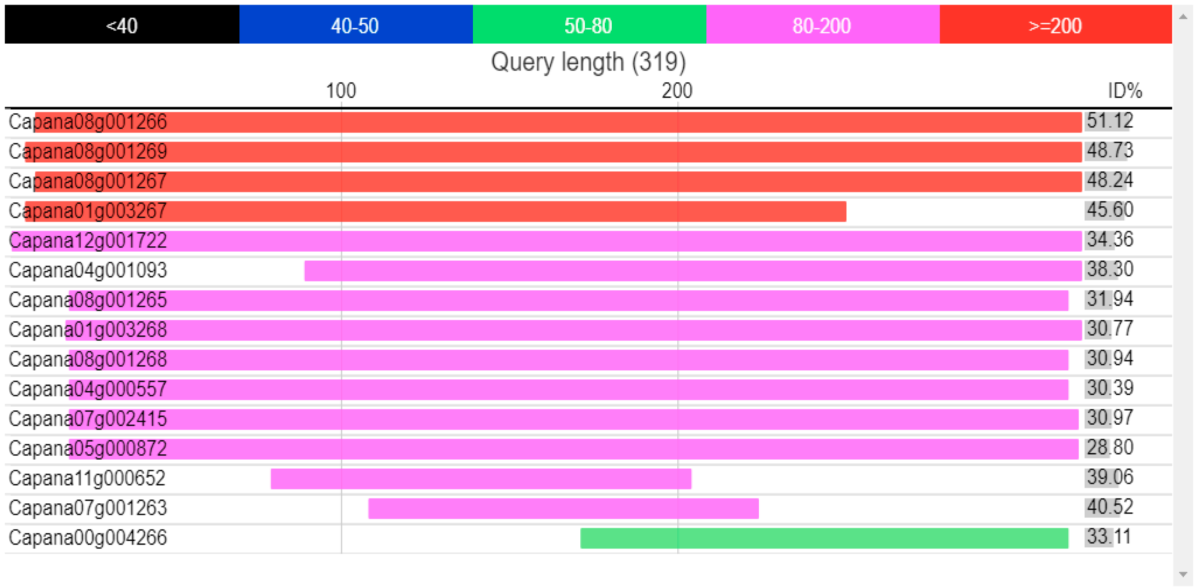

Figure S4. Homologous proteins with Arabidopsis thaliana AtAnn4 in pepper genome database. Homology comparison comes from pepper protein sequence databases zunla v2.0.
